# Effects of Inoculation on the Pearlitic Gray Cast Iron with High Thermal Conductivity and Tensile Strength

**DOI:** 10.3390/ma11101876

**Published:** 2018-10-01

**Authors:** Guiquan Wang, Xiang Chen, Yanxiang Li, Zhongli Liu

**Affiliations:** 1 School of Materials Science and Engineering, Tsinghua University, Beijing 100084, China; wgq15@mails.tsinghua.edu.cn (G.W.); yanxiang@tsinghua.edu.cn (Y.L.); 2 Key Laboratory for Advanced Materials Processing Technology, Ministry of Education, Beijing 100084, China; 3 School of Nuclear Equipment and Nuclear Engineering, Yantai University, Yantai 264005, China; liuzhonglimanoir@163.com

**Keywords:** thermal conductivity, tensile strength, inoculation, gray cast iron

## Abstract

With the aim of improving the thermal conductivity and tensile strength of pearlitic gray cast iron, the influence of inoculation on structure and properties was experimentally investigated. Three group of irons with similar compositions were inoculated by Zr-FeSi, Sr-FeSi, and SiC inoculants, respectively. The metallographic analysis was used to measure the maximum graphite length, primary dendrites amount and eutectic colonies counts. For a certain carbon equivalent, it was confirmed that the thermal conductivity of pearlitic gray cast iron has a direct correlation with the maximum graphite length while the tensile strength was influenced mainly by the primary dendrites amount. The optimal structure and highest thermal conductivity and tensile strength were obtained by Sr-FeSi inoculant. MnS particles act a pivotal part in modifying the structure of gray cast iron. It was found that providing nucleation sites both for graphite and primary austenite is important to promote the thermal conductivity and strength. However, excessive nuclei (MnS particles) results in shorter graphite flakes and thus the depressive growth of primary dendrites.

## 1. Introduction

Although gray cast iron (GCI) has been considered as the primary choice to produce vital engine components for many decades, the service life is not satisfied yet. Due to the alternating firing loads and rapid thermal cycles, the failure modes in these parts are high cycle fatigue and thermal-mechanical fatigue [1]. The resistances to the fracture of these GCIs are both closely related to tensile strength and thermal conductivity [2,3]. It is, thus, necessary to develop high performance cast irons (HPCI) with high thermal conductivity while maintaining the high tensile strength. 

As presented by Riposan [4], pearlitic gray cast iron, consisting of a fully pearlitic matrix and evenly distributed A-type graphite, is preferred due to its optimum comprehensive properties among all gray cast irons. However, there was a competitive relationship between the thermal conductivity and tensile strength of pearlitic GCI. Generally, the principal methods of approving tensile strength are to decrease the fraction of graphite flakes as well as to reduce their length [5,6]. On the contrary, the thermal conductivity can benefit from increasing of the graphite amount and graphite size [7]. It was reported that high tensile strength could be favored by developed primary dendrites [8], while a negative effect of the decrease of eutectic colonies size on the thermal conductivity was suggested due to a larger number of matrix discontinuities between graphite skeletons [9]. However, very few investigations have directly carried out to simultaneous promote both the thermal conductivity and tensile strength.

Inoculation has been considered as an effective method to improve the performance of GCI at low cost. It has been showed that Al, Ce, and Zr can increase the dendrites amount by directly influencing the nucleation of austenite [10]. Riposan found that Sr-FeSi was efficient in refining the eutectic colonies and nucleating the primary austenite [11]. As reported by Edalati, a homogeneous structure consisting of the uniform distribution of A-type graphite and increased eutectic colonies count could be obtained by SiC inoculation [12]. Nevertheless, limited work has been performed to study the effects of inoculation both on the microstructure and properties, especially thermal conductivity and tensile strength.

This work was undertaken to find out the possibility of the development of HPCI with a combination of high thermal conductivity and tensile strength through inoculation. Samples with various microstructure were produced using different inoculants, including Zr-FeSi, Sr-FeSi, and SiC inoculants. The influence of microstructural characteristics, mainly including of graphite length and primary dendrite percentage, was investigated to clarify the role of structural features in affecting both of thermal conductivity and strength. A detailed analysis of electronic microscopy was provided with particular attention given to the mechanism of the evolution of the microstructure.

## 2. Experimental Details

Nine gray cast iron ingots were melted in a 500 kg, medium-frequency induction furnace. The charge consisted of 70 wt% steel scrap and 30 wt% pig iron. Ferromolybdenum, ferromanganese, ferrosilicon and carburizer were used to meet the requirements of the composition. After superheating to 1530 °C, the liquid iron was transferred into a ladle and then poured into an EN-1561 Type II mould. Inoculants were deposited on the bottom of the ladle before pouring. The nominal composition of the samples and inoculants is given in Table 1. Carbon equivalent (CE) is calculated by the formula CE = C% + 0.31 Si% + 0.33 P%.

Tensile strength was measured using dog-bone shaped bars with 20 mm diameter in the gauge section, 60 mm gauge length and 3.2 μm surface finish according to Chinese Standard GB/T T228.1-2010. Three tests were performed for each composition and the average value was taken. Disk specimens with a diameter of 12.5 mm and a thickness of 2.5 mm were then cut from the grip section and used to determine the thermal diffusivity (α) and heat capacity ($c_{p}$) at room temperature using a NETZSCH LFA 457 laser flash apparatus (NETZSCH GABO instruments GmbH, Ahlden, Sachsen, Germany). The volume of the specimen was measured by the Archimedes method based on the fact that an object placed in a liquid displaces a volume of liquid equals to the volume of the object. The density (ρ) was obtained by weighting the specimen and dividing by the volume. And the thermal conductivity (*λ*) is calculated by:

(1) $$\lambda =\alpha \;c_{p}$$

Optical microscopy and field-emission scanning electron microscopy (SEM, JEOL, Akishima, Tokyo, Japan) equipped with an energy-dispersive X-ray spectroscopy (WDS) were used to analyze the microstructure. The length and area fraction of graphite were then evaluated in an unetched condition by quantitative metallography with the software Image J Pro (Version 6.0, Media Cybernetics, Rockville, MD, USA). The samples were then etched by 4% nital to expose the matrix phases. The eutectic colonies were revealed by the Stead Le Chatelier etchant (4 g MgCl_2_, 1 g CuCl_2_, 2 mL HCl, 100 mL alcohol). Color etching was also performed to evaluate the area fraction of primary austenite. The color etchant was 50 g NaOH and 4 g picric acid dissolved in 100 mL distilled water. The etching procedure was carried out at 98 °C for six minutes. The volume fraction of a phase is simply assumed as the area fraction occupied by the phase on the metallographic specimen. A total of eight fields were measured on each specimen’s cross-section.

## 3. Results

### 3.1. Metallographic Analysis

**Figure 1:**
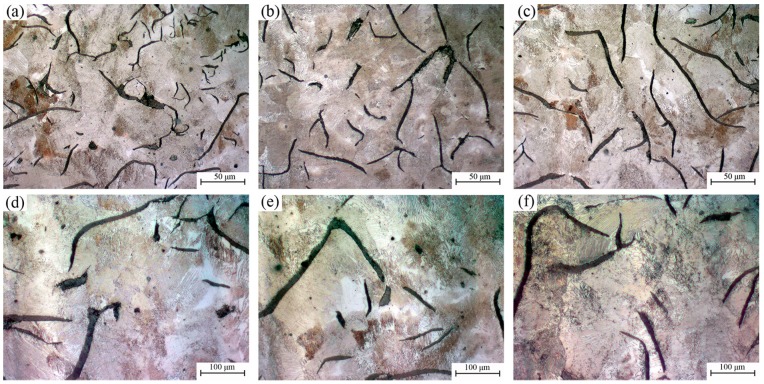
Optical images showing the matrix of samples inoculated by Zr-FeSi (**a**,**d**), Sr-FeSi (**b**,**e**), and SiC (**c**,**f**).

**Figure 2:**
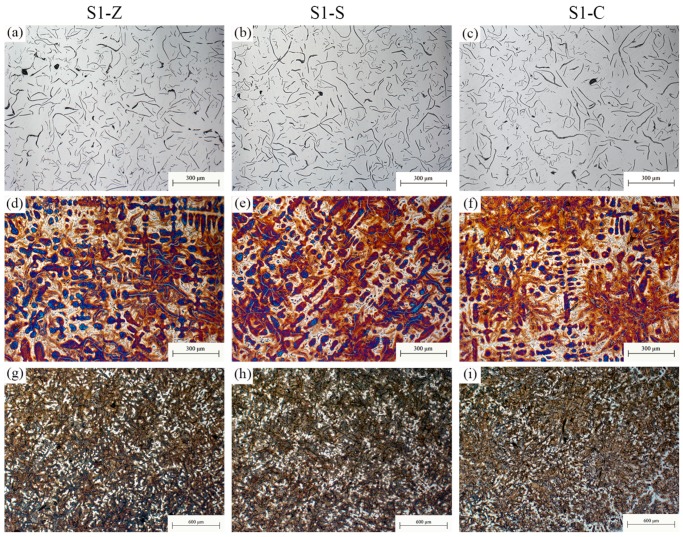
Metallographic images of S1-Z (**a**,**d**,**g**), S1-S (**b**,**e**,**h**) and S1-C (**c**,**f**,**i**), showing graphite (**a**–**c**), primary dendrite (**d**–**f**), and eutectic colonies (**g**–**i**).

**Figure 3:**
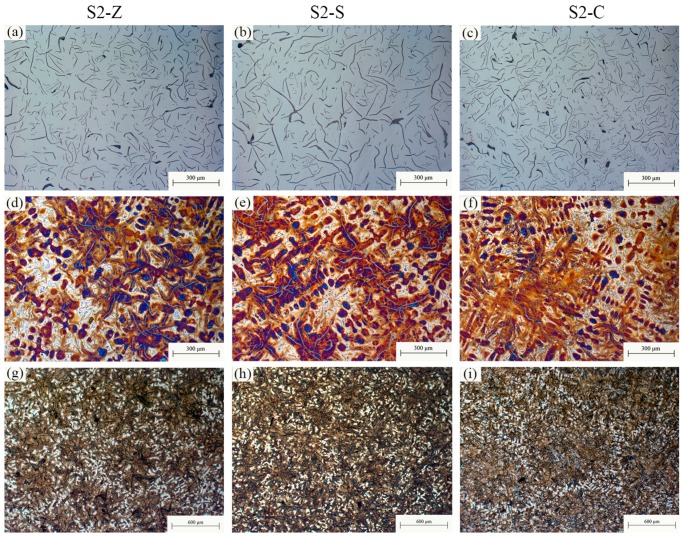
Metallographic images of S2-Z (**a**,**d**,**g**), S2-S (**b**,**e**,**h**) and S2-C (**c**,**f**,**i**), showing graphite (**a**–**c**), primary dendrite (**d**–**f**), and eutectic colonies (**g**–**i**).

**Figure 4:**
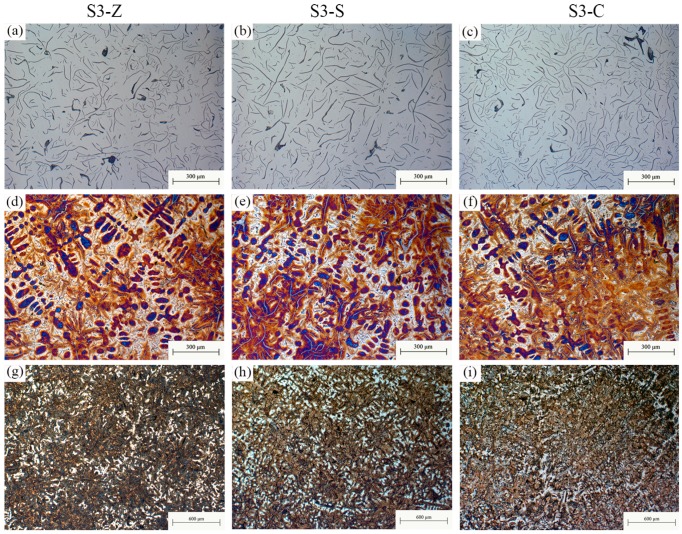
Metallographic images of S3-Z (**a**,**d**,**g**), S3-S (**b**,**e**,**h**) and S3-C (**c**,**f**,**i**), showing graphite (**a**–**c**), primary dendrite (**d**–**f**), and eutectic colonies (**g**–**i**).

**Figure 5:**
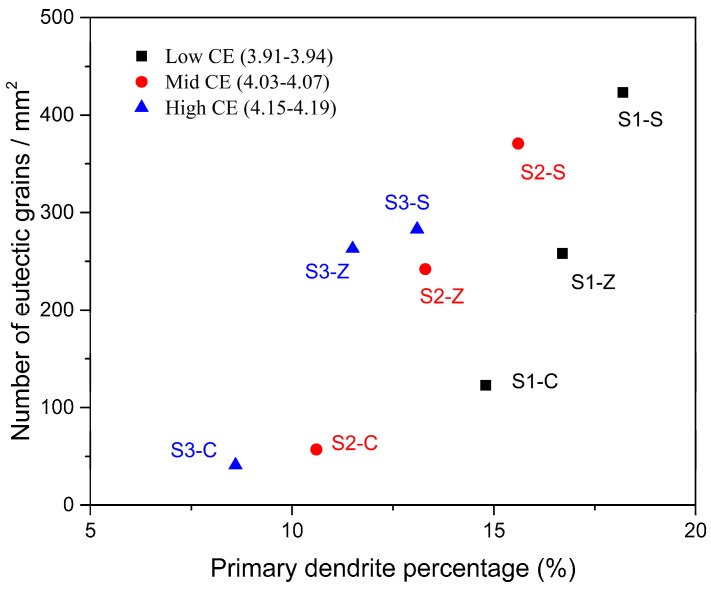
The primary dendrite percentage versus the number of eutectic colonies.

The typical images showing the matrix of irons inoculated by different inoculants are provided in Figure 1. According to GB/T 7216-2009, homogeneous structure consisting of a fully pearlitic matrix and evenly distributed A-type graphite was observed in all irons. Similar lamellar spaces of pearlite can also be found among different inoculations (as shown in Figure 1c,d). Other structural information of all investigated samples was shown in Figure 2, Figure 3 and Figure 4 according to similar CE. Corresponding microstructural characteristics were summarized in Table 2. The average of the three longest flakes in the field of view was taken as the maximum length since most of the graphite flakes are incomplete in a random 2D section. With the increase of CE, the increase of graphite content and decrease of primary dendrite amount were found. Additionally, no influence of inoculation on the graphite content was observed. The important differences between various inoculation mainly appear in the maximum flake length, primary dendrite percentage and eutectic colonies size. For a similar CE, the Zr-FeSi inoculant resulted in the shortest graphite flakes, moderate primary dendrite and moderate size of the eutectic colonies. The longest graphite length, the highest primary dendrite percentage and the smallest eutectic colonies size were found in the samples inoculated by Sr-FeSi. The maximum graphite length in SiC inoculated samples is similar to that in Sr-FeSi inoculated ones. Moreover, SiC inoculated the lowest dendrite amount and the biggest eutectic colonies. As shown in Figure 5, for similar CE (graphite percentage), a clear linear relationship between primary dendrite percentage and eutectic colonies was found in the present work. Therefore, the primary dendrite percentage and graphite size are mainly concerned in the subsequent analysis.

### 3.2. SEM Analysis

**Figure 6:**
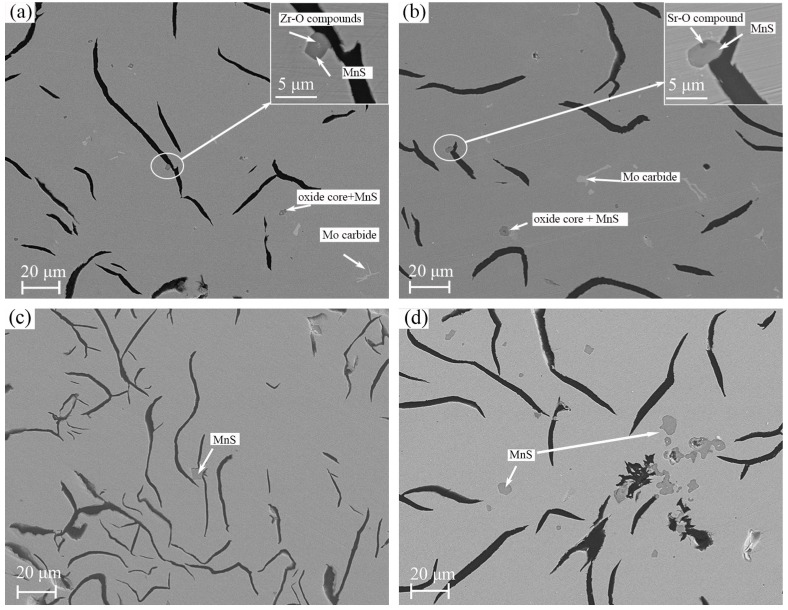
SEM images showing the morphology of MnS particles in S1-Z (**a**), S1-S (**b**), and S1-C (**c**,**d**).

Typical SEM microstructures treated by different inoculants were shown in Figure 6. MnS particles were found to be embedded in the matrix and in superficial contact with graphite in all the samples. Differences mainly appear in the morphology and the distribution of particles. X oxides (X = Zr or Sr) were found in the core of most MnS particles in X-FeSi inoculated samples, while no visible inclusions in MnS were observed in SiC inoculated ones. In SiC inoculated samples, large size, and clustered MnS particles were observed as shown in Figure 6c,d. On the contrary, a significant small and evenly distributed MnS were found in X-FeSi inoculated samples.

**Figure 7:**
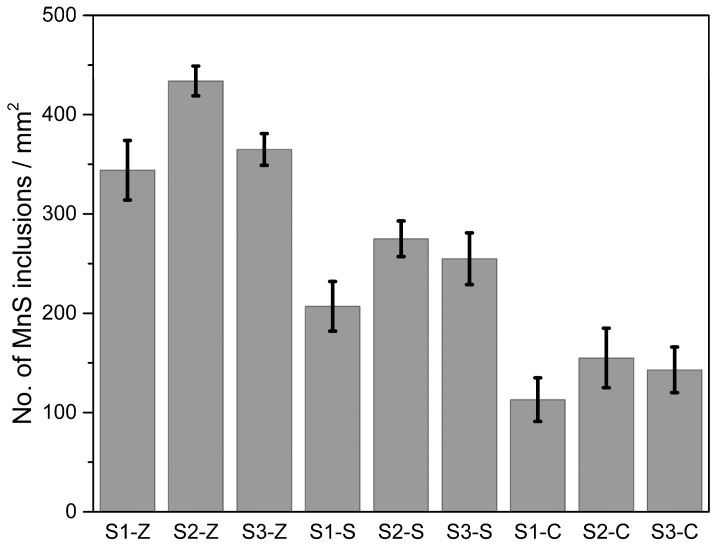
Statistical analysis of the quantity of MnS particles. Error bars are ± one standard deviation.

A statistical analysis was performed on the same samples to count the MnS particles. The average value of 8 SEM images at a random location of the polished surface of samples was recorded as the count. The results are provided in Figure 7. Remarkable differences were observed in the count of MnS particles depending on the selected inoculant. The Zr-FeSi inoculated samples have the largest number of MnS particles while the least number was found in SiC inoculated irons.

### 3.3. Tensile and Thermal Properties

**Figure 8:**
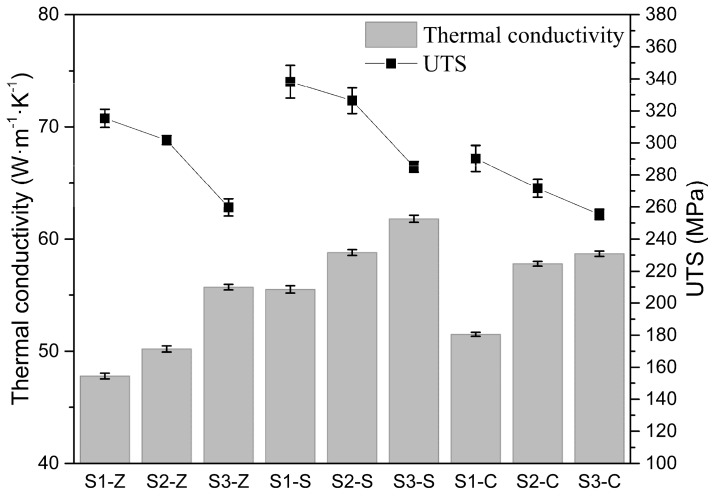
Tensile strength and thermal conductivity of all compositions. Error bars are ± one standard deviation.

The tensile strength and thermal conductivity of all compositions are provided in Figure 8. For a certain inoculation process, there is a clear negative correlation between tensile strength and thermal conductivity. However, for various inoculation, this relationship is untenable. The highest tensile strength and the highest thermal conductivity were achieved by the samples inoculated by Sr-FeSi. The Zr-FeSi inoculated irons have higher strength but lower thermal conductivity than that inoculated by SiC. It is suggested that improved tensile and thermal properties can be obtained simultaneously by good inoculation.

## 4. Discussion

It is well known that the properties of as-cast GCI are affected by chemical composition and inoculation. The graphite amount is mainly depended on the CE, which transforms the effect of elements on the graphite precipitation into relative content of carbon. As shown in Figure 8 and Table 2, the increasing of graphite amount has a clear positive effect on the thermal conductivity but a negative effect on the strength. It can be explained by the double-edge of graphite: improving the heat conduction but dissevering the matrix. However, for a wide range of CE, the inoculation in the current work clearly modifies the microstructure and thus change the thermal and tensile properties of GCI, as shown in Table 2 and Figure 8. 

**Figure 9:**
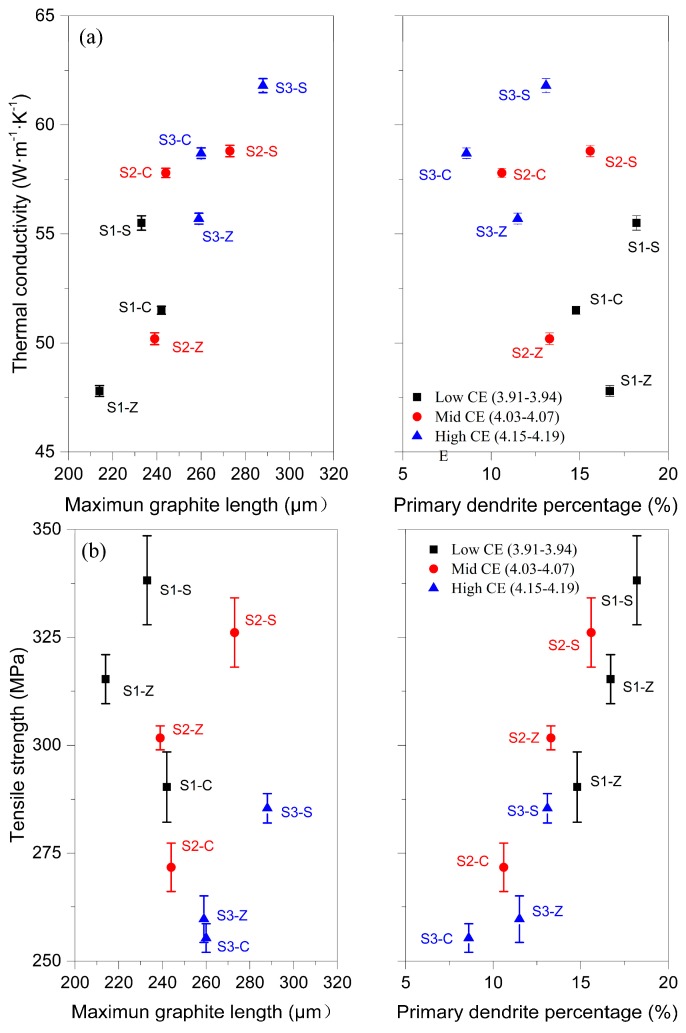
Relationship between the structural characteristics and properties: thermal conductivity (**a**) and tensile strength (**b**). Errors are ± one standard deviation.

The high tensile and thermal properties of Sr-FeSi inoculated GCI may result from more developed primary dendrites and longer graphite flakes. As presented in Figure 9a, the heat conductivity is found to increases with increasing maximum flake length. Although it was expected that an increased primary dendrite amount will reduce the thermal conductivity for the increasing matrix bridges over which the heat has to pass, such an effect was not established in the present work. The observed harmful effects of increasing primary dendrite amount on thermal conductivity for the same inoculation could be the results of decreasing CE and thus graphite amount. Contrary to thermal conductivity, the strength was mainly determined by the primary dendrite amount, as shown in Figure 9b. The more amount of primary dendrite, the higher strength. The impacts of graphite length are weakly, indecisive at least. The fact that tensile strength was largely determined by primary dendrite amount supports the theory that the eutectic colonies and the graphite flakes can extend over the primary arms without affecting the material strength.

As presented by Riposan, for FeSi containing deoxidizing elements X (X = Sr or Zr), X promotes the formation of small oxide micro-inclusions at high superheating temperature [13]. The precipitated oxides provide the substrate on which MnS can nucleate and grow. The so-called (Mn, X)S compound consisting of oxide and MnS provides lots of nuclei sites for primary austenite and eutectic graphite. For SiC [14], the inoculant dissolves into the melt through the reaction:

SiC + Fe → FeSi + C (dissociative)(2)

The generated dissociative carbon then provides the nucleation sites for graphite because of a high activity and zero mismatch. The observations as shown in Table 2 and Figure 6 support the theories mentioned above, which can explain why the fewest dendrites and the smallest number of MnS particles were observed in SiC inoculated alloys. 

Even though the Zr-FeSi inoculated samples have the largest number of MnS particles, which were considered as the effective nuclei for primary austenite and graphite, the most developed primary dendrites were found in Sr-FeSi inoculated GCI. The probable explanation can be provided by the arguments involving nucleation and growth kinetics of the phases. As reported by Rivera [15], in inoculated hypoeutectic melt solidification starts with the independent nucleation of austenite dendrites and graphite. When the dendrites grow and come into contact with the graphite as temperature drops, the units of lamellar graphite and austenite grow cooperatively and finally form the eutectic colonies. The larger amount of (Mn, Zr)S compounds in Zr-FeSi inoculated irons means more nuclei site for the austenite and graphite at the beginning of solidification and, thus, more opportunity to the interaction of primary austenite and graphite. As a result, the growth of primary dendrite is suppressed because of the earlier growth of the eutectic colonies. The fact that shorter graphite flakes and larger eutectic colonies were found in Zr-FeSi inoculated irons supports this assumption. It is suggested that the reason for the optimal structure of Sr-FeSi inoculated GCI is that Sr leads to moderate nucleation site density both for primary austenite and graphite.

## 5. Conclusions

For a similar CE, it was confirmed that the dominant structural factors in increasing thermal conductivity and tensile strength of pearlitic GCI are different. The thermal conductivity is determined by maximum graphite length while the tensile strength is mainly affected by primary dendrites amount. Long graphite flakes and developed dendrites are preferred for high thermal conductivity and strength.

In practice, the optimal structure can be obtained by good inoculation. While SiC additions inoculated long A-type graphite flakes, it did not appear to provide nucleation sites for primary austenite. Zr-FeSi and Sr-FeSi inoculated both primary austenite and graphite by promoting the nucleation of MnS at high temperature. However, the optimal structure and properties were found in Sr-FeSi inoculated irons. It is probably that Sr-FeSi inoculant provided the appropriate size and number of MnS particles. More dispersive MnS particles in Zr-FeSi inoculated irons resulted in shorter graphite and fewer dendrites amount because of excessively nucleation site.

## Figures and Tables

**Table 1:** The additives and chemical composition (wt%) of samples and inoculants.

Number	C	Si	Mn	P	S	Mo	Cu	Sn	CE	Additive
S1-Z	3.39	1.64	0.46	0.027	0.025	0.35	0.55	0.057	3.91	0.4 wt% Ino_1
S2-Z	3.53	1.59	0.51	0.029	0.028	0.34	0.54	0.060	4.03	0.4 wt% Ino_1
S3-Z	3.67	1.51	0.51	0.028	0.029	0.34	0.54	0.059	4.15	0.4 wt% Ino_1
S1-S	3.42	1.63	0.49	0.028	0.027	0.35	0.58	0.061	3.94	0.4 wt% Ino_2
S2-S	3.54	1.62	0.51	0.025	0.028	0.35	0.58	0.060	4.05	0.4 wt% Ino_2
S3-S	3.69	1.59	0.51	0.027	0.030	0.35	0.58	0.061	4.19	0.4 wt% Ino_2
S1-C	3.34	1.80	0.50	0.028	0.025	0.36	0.58	0.061	3.91	0.8 wt% Ino_3
S2-C	3.53	1.70	0.52	0.028	0.026	0.36	0.58	0.061	4.07	0.8 wt% Ino_3
S3-C	3.64	1.61	0.51	0.028	0.028	0.36	0.58	0.060	4.15	0.8 wt% Ino_3
Ino_1	Zr-FeSi (2.6 wt% Zr)									
Ino_2	Sr-FeSi (2.0 wt% Sr)									
Ino_3	SiC									

**Table 2:** Microstructural characteristics of samples. Errors are ± one standard deviation.

Sample No.	Graphite Type	Graphite Pct. (%)	Max. Length of Graphite (μm)	Primary Dendrite Pct. (%)	Eutectic Colonies Count (/cm^2^)
S1-Z	A	8.6 ± 0.5	214 ± 9	16.7 ± 0.7	258 ± 17
S2-Z	A	8.9 ± 0.4	239 ± 23	13.3 ± 0.6	242 ± 29
S3-Z	A	10.1 ± 0.6	259 ± 26	11.5 ± 0.6	263 ± 35
S1-S	A	8.5 ± 0.4	233 ± 13	18.2 ± 0.5	423 ± 29
S2-S	A	9.0 ± 0.2	273 ± 19	15.6 ± 0.9	371 ± 19
S3-S	A	9.9 ± 0.3	288 ± 25	13.1 ± 0.5	283 ± 22
S1-C	A	8.9 ± 0.3	242 ± 16	14.8 ± 0.6	123 ± 34
S2-C	A	9.4 ± 0.5	244 ± 20	10.6 ± 0.4	57 ± 19
S3-C	A	10.0 ± 0.2	260 ± 17	8.6 ± 0.5	42 ± 0

## References

[1] Langmayr F., Zieher F., Lampic M. (2004). The thermo-mechanics of cast iron for cylinder heads. MTZ Worldw..

[2] Seifert T., Riedel H. (2010). Mechanism-based thermomechanical fatigue life prediction of cast iron. Part I: Models. Int. J. Fatigue.

[3] Holmgren D., Svensson I.L. (2005). Thermal conductivity-structure relationships in grey cast iron. Int. J. Cast Met. Res..

[4] Riposan I., Chisamera M., Stan S. (2014). New developments in high quality grey cast irons. China Foundry.

[5] Collini L., Nicoletto G., Konecna R. (2008). Microstructure and mechanical properties of pearlitic gray cast iron. Mater. Sci. Eng. A.

[6] Xu W., Ferry M., Wang Y. (2005). Influence of alloying elements on as-cast microstructure and strength of gray iron. Mater. Sci. Eng. A.

[7] Helsing J., Grimvall G. (1991). Thermal-Conductivity of Cast-Iron—Models and Analysis of Experiments. J. Appl. Phys..

[8] Fourlakidis V., Dioszegi A. (2014). A generic model to predict the ultimate tensile strength in pearlitic lamellar graphite iron. Mater. Sci. Eng. A.

[9] Ormerod J., Taylor R., Edwards R.J. (1978). Thermal-Diffusivity of Cast Irons. Met. Technol..

[10] Chisamera M., Riposan I., Stan S., Militaru C., Anton I., Barstow M. (2012). Inoculated Slightly Hypereutectic Gray Cast Irons. J. Mater. Eng. Perform..

[11] Riposan I., Chisamera M., Stan S., Skaland T. (2003). Graphite nucleant (microinclusion) characterization in Ca/Sr inoculated grey irons. Int. J. Cast Met. Res..

[12] Edalati K., Akhlaghi F., Nih-Ahmadabadi A. (2005). Influence of SiC and FeSi addition on the characteristics of gray cast iron melts poured at different temperatures. J. Mater. Process. Technol..

[13] Riposan I., Chisamera M., Stan S., Hartung C., White D. (2010). Three-stage model for nucleation of graphite in grey cast iron. Mater. Sci. Technol..

[14] Xue W.D., Li Y. (2016). Pretreatments of gray cast iron with different inoculants. J. Alloy Compd..

[15] Rivera G., Calvillo P.R., Boeri R., Houbaert Y., Sikora J. (2008). Examination of the solidification macrostructure of spheroidal and flake graphite cast irons using DAAS and ESBD. Mater. Charact..

